# Anatomically informed deep learning framework for generating fast, low-dose synthetic CBCT for prostate radiotherapy

**DOI:** 10.1038/s41598-025-23781-7

**Published:** 2025-10-15

**Authors:** Mustafa Kadhim, Emilia Persson, André Haraldsson, Christian Jamtheim Gustafsson, Mikael Nilsson, Malin Kügele, Sven Bäck, Sofie Ceberg

**Affiliations:** 1 Department of Medical Radiation Physics, Lund University, Lund, Sweden; 2 Radiation Physics, Department of Hematology, Oncology, and Radiation Physics, Skåne University Hospital, Lund, Sweden; 3 Medical Radiation Physics, Department of Translational Medicine, Lund University, Malmö, Sweden; 4 Centre for Mathematical Sciences, Lund University, Lund, Sweden; 5 Department of Radiooncology, Rostock University Medical Center, Rostock, Germany

**Keywords:** Cancer imaging, Computer science, Imaging techniques, Prostate

## Abstract

Precise patient positioning and daily anatomical verification are crucial in external beam radiotherapy to ensure accurate dose delivery and minimize harm to healthy tissues. However, Current image-guided radiotherapy techniques struggle to balance high-quality volumetric anatomical visualization and rapid low-dose imaging. Addressing this, reconstructing volumetric images from ultra-sparse X-ray projections holds promise for significantly reducing patient radiation exposure and potentially enabling real-time anatomy verification. Here, we present a novel DL-based framework that generates synthetic volumetric cone-beam CT in real-time from two orthogonal projection views and a reference planning CT for prostate cancer patients. Our model learns the mapping between 2D and 3D domains and generalizes across patients without retraining. We demonstrate that our framework produces high-fidelity volumetric reconstructions in real-time, potentially supporting clinical workflows without hardware modifications. This approach could reduce imaging dose and treatment time while preserving comprehensive anatomical information, offering a pathway for safer, more efficient prostate radiotherapy workflows.

**Supplementary Information:**

The online version contains supplementary material available at 10.1038/s41598-025-23781-7.

## Introduction

Prostate cancer is among the most frequently diagnosed malignancies in men worldwide^1^, and external radiotherapy plays an important role in managing its progression and can be employed for curative, salvage, and palliative treatments^2–5^. A precise geometric delivery of therapeutic doses to the clinical target volume (CTV) while minimizing dose exposure to adjacent healthy organs at risk (OARs) is essential to reduce treatment-related toxicities, enhance tumor control, and lower the risk of recurrence^6,7^.

A persistent challenge in prostate cancer radiotherapy is organ motion, being either inter- or intra-fractional motion, which represents anatomical displacements caused by physiological processes such as bladder or rectal filling, and muscle tension or relaxation before and during treatment delivery^8–10^. Such motion may lead to insufficient tumor coverage or unintended radiation exposure of adjacent OARs^11–13^. Moreover, longer treatment session durations have been shown to increase the probability of intra-fractional motion^14,15^.

In addition to organ motion, day-to-day variations in patient setup and equipment limitations can introduce residual setup uncertainties that require to be accounted for during treatment planning^16^. To address these uncertainties in clinical practice, a planning target volume (PTV) is defined by adding a geometric safety margin to the CTV^17–19^. However, while creating a PTV ensures target coverage, it also increases the volume of healthy tissue exposed to radiation.

With the use of image guided radiotherapy (IGRT) setup uncertainties can be corrected and the size of PTV can in theory be reduced, resulting in decreased treatment toxicity while maintaining sufficient tumor control^20,21^.

Current IGRT techniques for prostate cancer on C-arm linear accelerators (LINACs) commonly include acquiring three-dimensional (3D) kilovolt cone-beam computed tomography (CBCT) images, or two-dimensional (2D) orthogonal kilovoltage (kV) X-ray images^22,23^. CBCT images provide detailed 3D visualization of the internal anatomy, facilitating accurate verification of patient setup and target al.ignment. However, CBCT imaging is limited by high operational cost, susceptibility to artifacts, non-negligible imaging doses to patients (up to 31.5 mGy/fraction^24,25^, and slow image acquisition^26^. As patients undergo numerous CT and CBCT scans throughout their treatment, the cumulative radiation dose both at and around the target area gradually increases, thereby increasing the risk of developing secondary cancers^27^. Additionally, the ALARA (As Low As Reasonably Achievable) radiation protection principal advocates minimizing radiation exposure to only what is clinically necessary^28^, encouraging the use of alternative imaging methods when possible.

Furthermore, extended imaging durations prolong the treatment sessions, which may increase patient discomfort and increase the risk of intra-fraction motion^10^. In standard IGRT workflows, the CBCT acquisition can take up to one minute, while prostate displacement may occur within a few seconds^29^. This displacement can result in a blurred or inaccurate target localization in the acquired setup images.

In contrast, 2D-kV imaging is fast (high temporal resolution) and yields substantially less imaging dose (a reduction by a factor of 3-12 ×^30^ compared to CBCT. For prostate cancer patients, the 2D-kV images are commonly acquired orthogonally to the treatment beam (anterior-posterior and lateral directions) and used to align bony structures or implanted fiducial markers. These images can also be acquired during radiation delivery for near real-time motion monitoring. However, 2D-kV images create anatomical ambiguity by collapsing 3D structures into a single plane. This superposition of overlapping tissues prevents the reliable verification of soft tissue targets like the prostate and OARs (e.g. bladder and rectum). For patient alignment, the 2D-kVs are routinely compared and aligned with digitally reconstructed radiographs (DRRs) generated from treatment pCT.

The tradeoff between 2D-kV and CBCT images underscores a critical unmet need in current IGRT techniques, the need for fast, low-dose imaging that offers comprehensive anatomical visualization in 3D. To tackle this challenge, several studies have investigated deep learning (DL) methods to inversely reconstruct 3D volumetric images from single or multiple 2D projections^26,31–38^, with the aim of enabling comprehensive soft tissue visualization without increasing imaging dose. To our knowledge the investigation of such DL frameworks for prostate cancer patients remains unexplored. Additionally, to mitigate data scarcity, 2D-DRR images generated from pCT are often used as substitutes to mimic real 2D-kV images for DL model development^34,39–43^. Moreover, previous DL frameworks^26,32–44^ have not explored the feasibility of utilizing an additional imaging modality to use as a reference for patient anatomy during model development. Such as utilizing both the 2D-DRRs and pCT images as dual inputs to the DL model to potentially generate more accurate synthetic volumetric images of the patient anatomy. Furthermore, the training and development of such models have also relied heavily on using standard pixel-wise or hybrid (combination of pixel-wise and human like perceptual similarity) loss functions without incorporating anatomically specific context that could be of importance during clinical decision-making. Thus, restricting the models from training with an anatomically informed loss function.

In this study, an anatomically informed DL model capable of rapidly generating volumetric synthetic CBCT (sCBCT) images using two daily orthogonal 2D-DRR projections and a reference 3D pCT was developed. Our model generates volumetric sCBCT within 8 milliseconds (ms). We also demonstrate the importance of using an anatomically informed loss function (ALF) to improve reconstruction of soft tissues at clinically relevant regions. The model performance was assessed through both qualitative assessment and quantitative image quality metrics. Our inference results demonstrated high similarity between the sCBCT and ground truth CBCT images, especially in clinically relevant regions. Our framework has the potential to reduce the reliance on unnecessary frequent CBCT acquisitions and enable real-time anatomical verification in prostate cancer radiotherapy.

## Results

### Framework overview

**Fig. 1:**
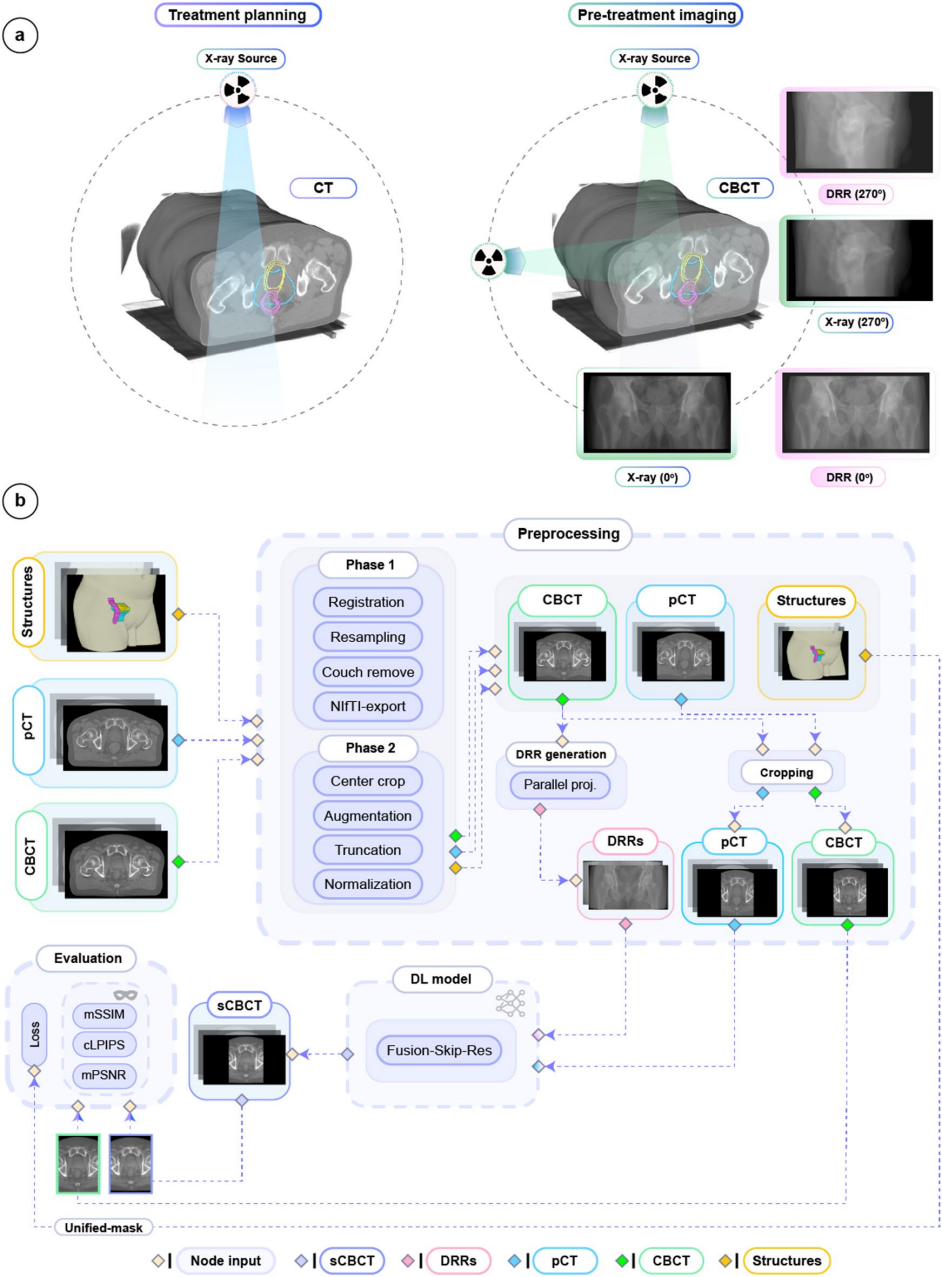
Overview of data types and framework. (**a**) Comparison of standard imaging procedures in prostate cancer radiotherapy. Left: Volumetric CT image acquisition to define target volumes and organs at risk used for accurate treatment planning. Overlayed on pCT are key radiotherapy structures being: PTV (teal), bladder (yellow) and rectum (magenta). The PTV delineates the area receiving 95% of the prescribed radiation dose. Right: Volumetric CBCT and/or orthogonal X-ray images that could be acquired for daily image-guided radiotherapy to verify prostate cancer patient setup and anatomy. The orthogonal images are acquired consecutively by rotating the machine gantry to each angle. Radiotherapy structures are overlaid on the CBCT to highlight the relevant anatomical regions considered during decision making at treatment delivery. (**b**) Flowchart of the data preprocessing, model inputs/output, and proposed model evaluation workflow. Dashed arrows indicate information flow through various stages of the framework. For each patient, radiotherapy structures (yellow box), pCT (blue box), and CBCT (green box) images were collected. In phase 1 of data preprocessing, all images underwent rigid registration, resampling, exclusion of non-body regions (couch remove), and conversion to NIfTI format. In phase 2, online (on the fly) preprocessing with the MONAI^45^ framework was implemented to center crop, augment (e.g., translations, rotations), truncate intensity between [-1000, 2000] Hounsfield units (HU), and normalize the images using per case min-max normalization to be between [0,1]. Further preprocessing details are provided. The PTV, bladder and rectum structures were converted into binary masks and combined to form a unified binary mask (Unified-mask). The Unified-mask was exclusively used during model training to serve as input to our hybrid anatomically informed loss function, ALF. The DRR images were generated via parallel projections from augmented CBCTs immediately after phase 2. The DRRs and cropped pCTs served as inputs to the DL model (Fusion-Skip-Res). The predicted sCBCTs (purple) along with their corresponding ground truth CBCTs (green) were used for loss computations and evaluation using masked mMAE, mSSIM, mPSNR, and cLPIPS. The body contour (beige) was used during preprocessing (**b**) and defined the region for calculating the masked image quality metrics.

We included a retrospective cohort of patients treated at Skåne University Hospital in Lund between 2019 and 2025, who received either 70 Gy in 35 fractions to the prostate bed or 36 Gy in 6 fractions to the prostate. Patients with hip implants, urinary catheter, or fiducial markers were excluded. In total, 419 prostate cancer patients with 419 pCTs, 3561 pre-treatment CBCTs, and 7122 orthogonal DRR images were included. Each patient had structures including target (PTV), and OARs (bladder, rectum, and body) delineated on their pCT (Fig. 1a). A flowchart of data preprocessing, model inputs/outputs, and selected performance evaluation metrics is presented in Fig. 1b.

Moreover, for our image reconstruction task we focus on accurately reconstructing anatomical regions of importance for decision-making at a pre-treatment verification step (CBCT acquisition step in clinical workflow). Hence, all the presented and used images in this framework were cropped to exclude irrelevant details outside these regions. For robust model evaluation, we used masked mean absolute error (mMAE), masked structure similarity index measure (mSSIM), masked peak signa-to-noise ratio (mPSNR), and cropped learned perceptual image patch similarity (cLPIPS). Here, we crop LPIPS to facilitate image patch calculations. The masking and cropping of metrics exclude the background (full of zeros) during model evaluation, to only evaluate the details within the body structure.

### Model evaluation and reconstruction performance

**Fig. 2:**
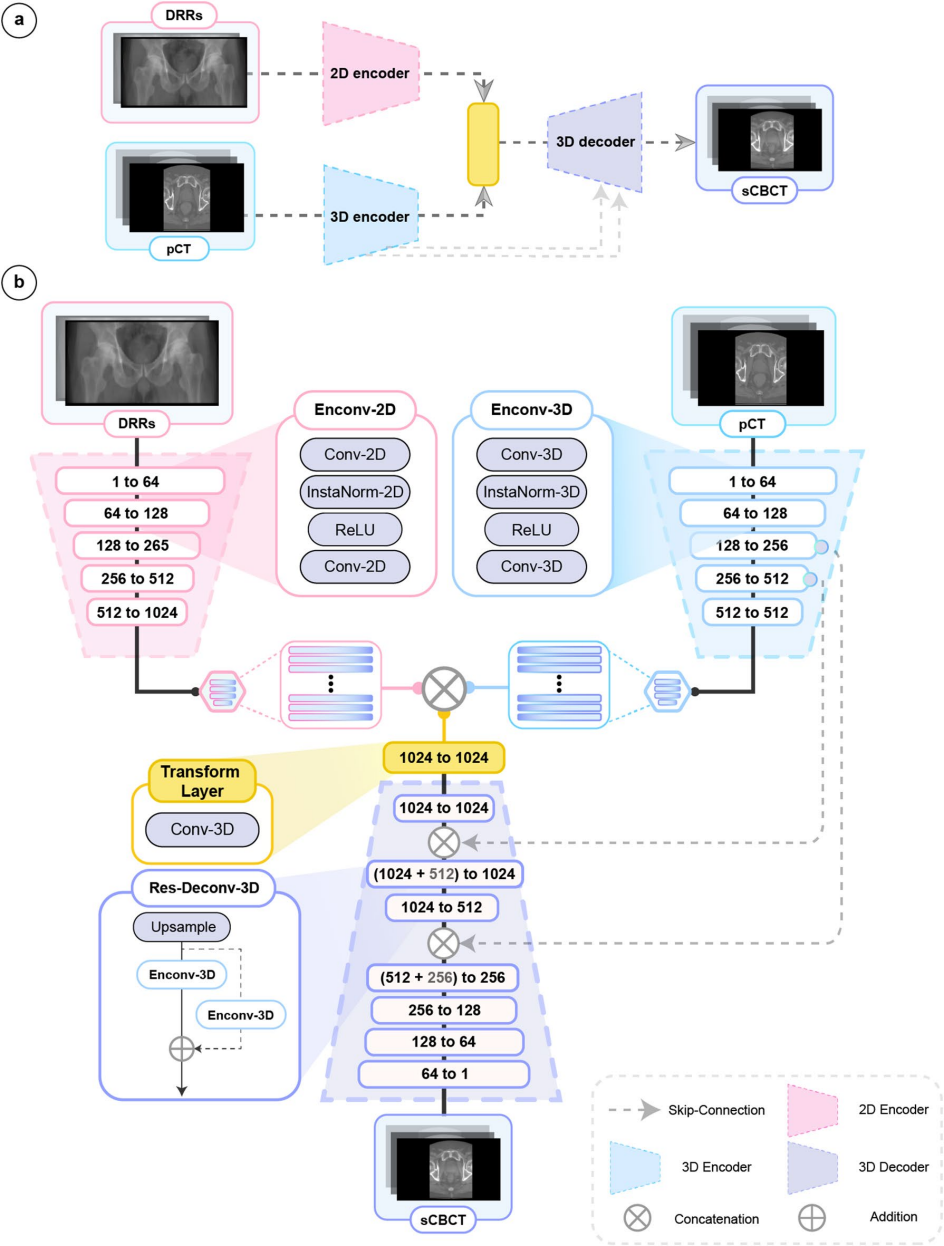
Overview of the Fusion-Skip-Res model. (**a**) High-level overview of the dual-branch encoder-decoder model architecture. The model processes orthogonal 2D-DRR projections through a 2D encoder (pink block) branch and the 3D planning CT (pCT) through a separate 3D encoder (blue block), fuses the resulting features into a transformation layer (yellow block), and reconstructs the synthetic CBCT (sCBCT) using a 3D decoder (purple block). Skip connections (grey dashed arrows) link the 3D encoder and decoder branches. (**b**) Detailed schematic of the model architecture consisting of 2D and 3D encoder blocks (Enconv-2D and Enconv-3D), a 3D transformation layer (Transform Layer, yellow), 3D residual upsampling layers (Res-Deconv-3D, purple), and skip connections (grey dashed arrows). The 2D encoder (pink) processes DRRs using a series of Enconv-2D blocks, each containing an initial downsampling 2D convolutional layer (stride 2), 2D instance normalization, ReLU activation, and a subsequent 2D convolutional layer (stride 1). The 3D encoder (light blue) processes the pCT using analogous Enconv-3D blocks with 3D operations. Feature map dimensions are indicated at each stage (e.g., 1 to 64 channels). The latent features from both encoders are concatenated and passed through the 3D transformation layer (yellow) with a 1 × 1 × 1 kernel size. The 3D decoder (purple) reconstructs the sCBCT volume using a series of upsampling blocks (Res-Deconv-3D), which incorporate trilinear upsampling and residual connections (addition symbol) around Enconv-3D layers. Skip connections (grey dashed arrows) concatenate features from the 3D encoder to corresponding 3D decoder levels.

A specialized DL model was developed and optimized for sCBCT reconstruction from dual orthogonal DRR projections and pCT images based on an encoder-decoder framework (see Fig. 2). The model employs a fusion mechanism to combine the extracted features from the 2D-DRRs with the 3D pCT scans and decode this information to generate volumetric sCBCT. Further details regarding the model building blocks and computer code are available below (see Model architecture, Methods, and for code, see Code availability). After systematic model design exploration, we identified that incorporating both skip and residual connections within the fusion model achieved optimal reconstruction quality and anatomical accuracy. This model is defined as Fusion-Skip-Res (Figs. 1b and 2).

To ensure robust training and evaluation, the model was trained using a patient-wise splitting strategy to prevent data leakage between training and evaluation sets. The model was trained on 355 patients (comprising 355 pCT volumes, 2728 CBCT volumes, and 5456 DRR projections), validated on 28 patients (pCT: 28, CBCT: 206, DRRs: 412), and tested on 32 patients (pCT: 32, CBCT: 627, DRRs: 1254) for final model performance assessment.

**Fig. 3:**
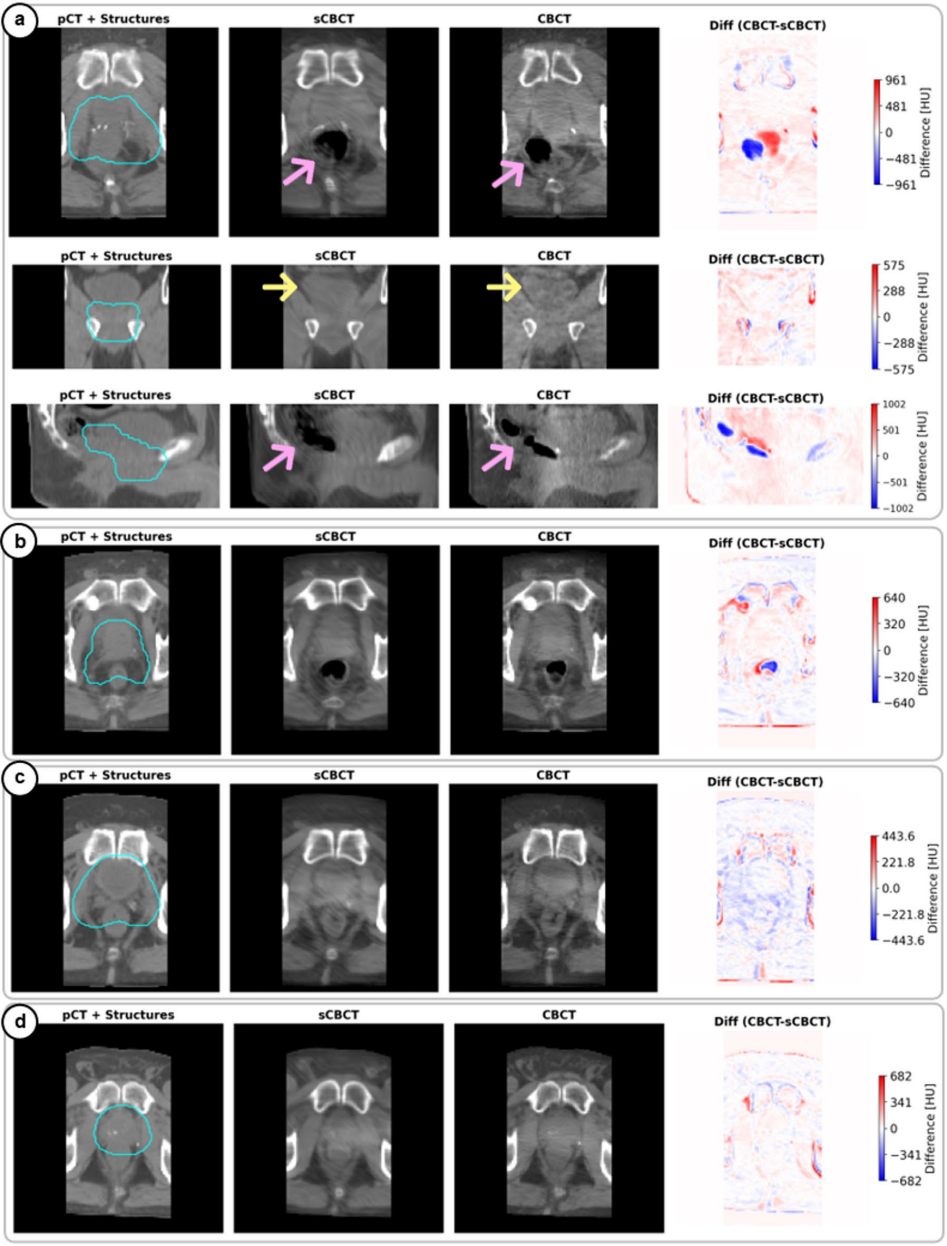
Reliable sCBCT reconstruction of daily anatomical changes. Performance of the Fusion-Skip-Res model on samples from the test set (**a**–**d**), where (**a**), Multi-plane visualization (axial, coronal, sagittal views from top to bottom) for a representative test patient showing planning CT (pCT) with planning target volume (PTV) (teal) structure as overlay (first column), synthetic CBCT (sCBCT) reconstructed by Fusion-Skip-Res model (second column), ground truth CBCT (third column), and the difference map (Diff) between ground truth and sCBCT given in Hounsfield units (HU) (fourth column). The PTV represents where 95% of the treatment dose is prescribed, the pink arrows indicate rectal filling variations, and yellow arrows highlight bladder volume changes. Additionally, note the accurate reconstruction of clinically significant anatomical changes that occurred between pCT and treatment day. (**b**–**d**), Axial views from three additional test patients demonstrating consistent reconstruction performance across varying anatomical cases. The difference maps reveal that reconstruction errors remain minimal within the PTV region and primarily occur at tissue-air interfaces and in certain bony edges distant from the treatment target. The average inference time (excluding data loading and GPU warmup) to generate each volumetric sCBCT was 8 ± 0.2 ms per patient. To preserve space and emphasize the fine anatomical structures in this figure, the input DRR images have been presented in figure (Fig. 4a).

**Fig. 4:**
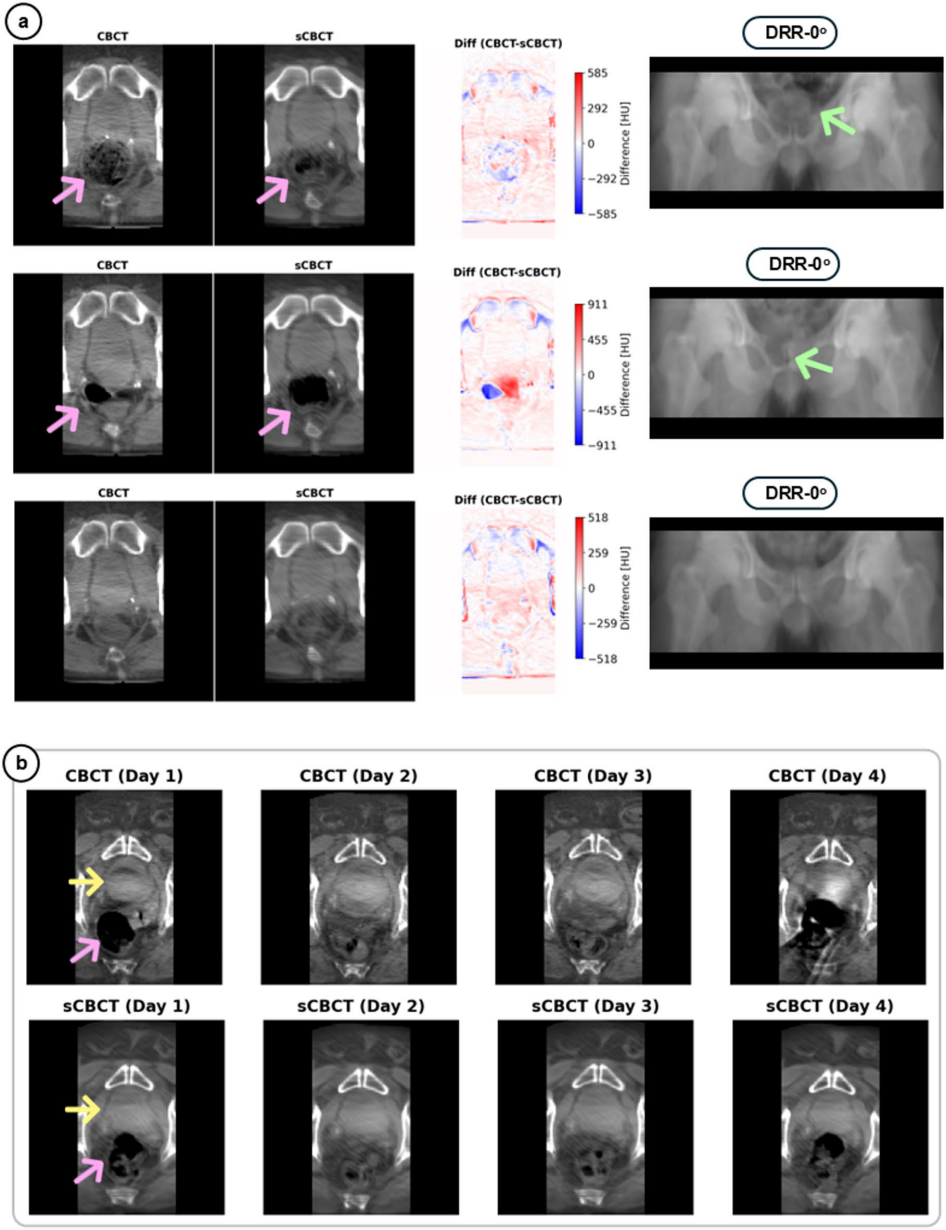
Reliable inter-fractional representation across treatment days. (**a**) Three consecutive treatment sessions (rows) for a single test patient showing ground truth CBCTs (left), synthetic CBCTs (sCBCT) (middle), difference maps between the CBCT and sCBCT images (right), and corresponding anterior DRR (far right) used as model input. Pink arrows indicate rectal filling variations captured in both reconstructions. Green arrows in the DRRs highlight how these anatomical changes manifest in the 2D projections. Note how the model accurately reconstructs these variations solely from the DRR information, with difference maps showing minimal reconstruction errors in clinically relevant regions except for air-tissue intersections. In (**b**) a challenging case demonstrating large inter-fractional anatomical changes across four treatment days. Top row: ground truth CBCT images showing substantial variations in bladder volume (yellow arrows) and rectal filling (pink arrows). Bottom row: corresponding sCBCT indicating changes in anatomical states, demonstrating the model’s robustness to significant inter-fractional changes.

The Fusion-Skip-Res model demonstrated high reconstruction accuracy in clinically relevant anatomical regions, reliably capturing inter-fractional variations in the target, bladder, and rectum—structures critical for accurate treatment delivery (Figs. 3 and 4). As observed, the anatomical information in sCBCT is not being directly duplicated from the pCT images (Fig. 3) but rather fused with the anatomical changes manifested in DRR images to generate more reliable sCBCT images (Fig. 4a). The generation time for each sCBCT using the Fusion-Skip-Res model took approximately 8 ms (excluding data loading and GPU warmup), which for reference is about 7500 times faster than conventional CBCT acquisition (~ 1 min). The model also offered the best results across all image similarity metrics compared to other explored models in Supplementary information (see Table 1, Tables).

**Table 1:** The average reconstruction performance of the explored models across the validation and test datasets using ALF as loss function.

Model type	Validation (*n* = 206)				Test (*n* = 627)			
	MAE [HU] (↓)	SSIM (↑)	PSNR [dB] (↑)	LPIPS (↓)	MAE [HU] (↓)	SSIM (↑)	PSNR [dB] (↑)	LPIPS (↓)
DRRs-only	110.2	0.71	28.60	0.11	109.4	0.79	26.06	0.21
Fusion	70.32	0.85	29.73	0.10	80.21	0.83	29.69	0.19
Fusion-Skip	79.64	0.84	31.86	0.07	67.65	0.83	30.84	0.13
Fusion-Skip-Res	**50.91**	**0.89**	**31.90**	**0.07**	**51.35**	**0.85**	**30.93**	**0.10**

The evaluation includes both qualitative and quantitative assessments, being masked mean absolute error (mMAE), masked structural similarity index (mSSIM), masked peak signal-to-noise ratio (mPSNR), and cropped learned perceptual image patch similarity (cLPIPS) metrics. The best results are highlighted in bold.

The model successfully learns from DRR images to incorporate daily anatomical changes into its 3D sCBCT outputs, where rectal variations manifested in the 2D-DRRs (Fig. 4a, green arrows) are faithfully reproduced in the sCBCT images (Fig. 4a, pink arrows). Furthermore, to assess temporal consistency in capturing inter-fraction anatomical changes, we analysed the model’s performance across consecutive treatment fractions (Fig. 4b). The model could reliably represent rectum and bladder changes based on the information provided by DRR images, changes that could impact dose delivery to the target and OARs. In a more challenging case (Fig. 4b), where a patient exhibited large inter-fractional variations in rectum (pink arrows) and bladder (yellow arrows), the model maintained high reconstruction fidelity across different treatment fractions.

### Influence of loss function on image quality

**Fig. 5:**
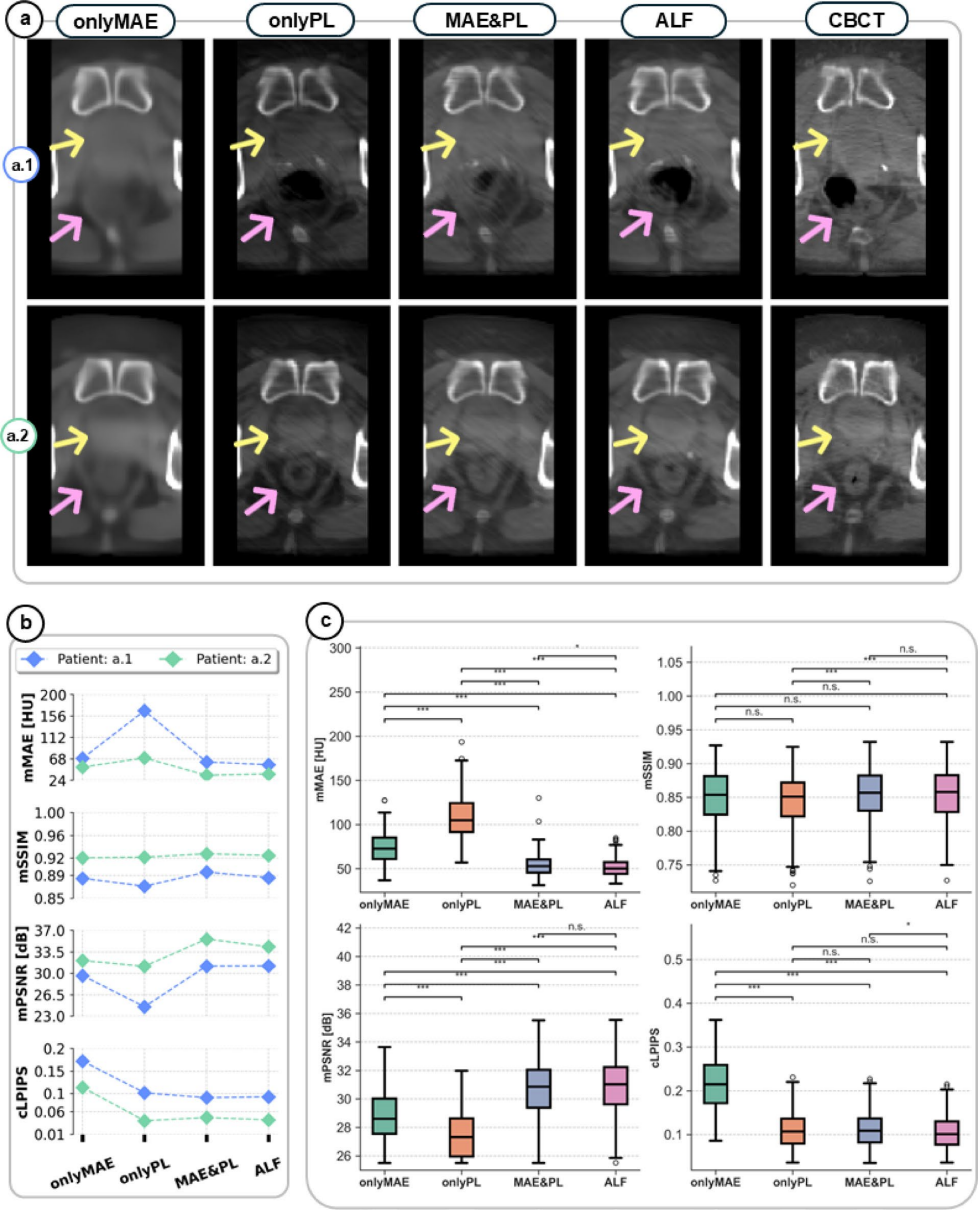
ALF loss function enables superior reconstruction quality. (**a**) Comparison of sCBCT reconstructions using different loss functions (columns) for two representative test patients (rows, a.1 and a.2). From left to right, the model training utilized: only a mean absolute error loss (onlyMAE), only a perceptual loss (onlyPL), a hybrid MAE and PL loss (MAE&PL), only the anatomy-informed loss function (ALF), and the ground truth CBCT given for comparison. Note the adequate soft tissue contrast and anatomical boundary definition achieved with ALF, particularly in regions critical for radiotherapy targeting such as bladder (yellow arrows) and rectum (pink arrows). The selected weighting factors for ALF during model training are presented in (Table 2, Tables). (**b**) Quantitative evaluation of loss function influence on image metrics for patients a.1 (blue) and a.2 (green), demonstrating ALF’s either superior or comparable performance across masked MAE (mMAE), masked structure similarity index (mSSIM), masked peak signal-to-noise ratio (mPSNR), and cropped learned perceptual image patch similarity (cLPIPS). Lower values are better for mMAE and cLPIPS; higher values are better for mSSIM and mPSNR. **c**) Distribution of image quality metrics across the entire test dataset (*n* = 627 CBCT-sCBCT volumes), showing consistent and in some cases significant improvements with ALF compared to other loss functions. Box plots show median (center line), 25th-75th percentiles (box limits), and 1.5× interquartile range (whiskers); outliers appear as individual points. Each box color represents a different loss function: onlyMAE (green), onlyPL (orange), MAE&PL (blue), and ALF (pink). Statistical significance is indicated as: *p < 0.05, **p < 0.01, and ***p < 0.001, and n.s. (not significant), determined using Kruskal-Wallis test followed by Dunn’s test with Bonferroni correction.

To benchmark our proposed anatomical loss function, ALF, against MAE and PL reconstruction loss functions, we conducted ablation experiments using the Fusion-Skip-Res model. The model was trained with four different loss functions: (i) only with MAE (onlyMAE), (ii) only with perceptual loss (onlyPL), (iii) with a hybrid loss combining both MAE and PL (MAE&PL), and (iv) only with ALF. Detailed definitions of these loss functions and their components are provided below (see Loss functions, Methods, and Ablation experiment, Methods). The reconstruction performance was assessed on the test dataset (Fig. 5a–c). Furthermore, Fig. 5a, b highlights the qualitative and quantitative impacts of selecting a proper loss function for model development, and Fig. 5c presents the quantitative impact of loss function selection across all test samples. Notably, ALF significantly reduced the reconstruction error (mMAE) by approximately 50% (*p* < 0.001) while maintaining superior or comparable performance in perceptual quality (mPSNR and cLPIPS) compared to onlyMAE and onlyPL and MAE&PL loss functions, respectively. Statistical analysis was performed using Shapiro-Wilk test for non-normality assessment and followed by Kruskal-Wallis test and a post-hoc Dunn’s test with Bonferroni correction. Moreover, the obtained significant improvements with ALF were achieved without increasing computational complexity during inference.

**Table 2:** The selected hyper-parameters for each ablation experiment conducted to determine the impact of loss function selection on DL model performance (Fig. 5; Results, and loss functions; Methods).

Loss function				
	$\:{\alpha\:}$	$\:{\beta\:}$	$\:{\gamma\:}$	$\:{\omega\:}$
MAE-only	1	0	0	0
PL-only	0	1	0	0
MAE&PL	1	0.02	0	0
ALF	1	0.05	0.01	0.04

## Discussion

Our study introduces a DL framework that rapidly generates volumetric synthetic CBCT images for prostate cancer patients using both orthogonal 2D-DRR images and planning CT as input. This approach reduces the gap between fast, low-dose 2D imaging and comprehensive 3D anatomical verification, representing a promising advancement for image-guided radiotherapy. We demonstrated that our Fusion-Skip-Res can produce sCBCT images that may be usable as an alternative to conventional CBCT, capturing daily anatomical changes faster than conventional CBCT acquisitions (~ 1 min).

The ability to capture daily volumetric anatomical information from 2D projections, without full CBCT acquisition, offers several important clinical advantages. First, it substantially reduces imaging-related radiation exposure (aligning with ALARA principles) while maintaining the ability to visualize 3D anatomical changes. This is particularly relevant for prostate cancer patients who undergo multiple imaging procedures throughout their treatment course, where the risk of developing secondary cancers (genitourinary and lung) increases with increased radiation exposure^46,47^. For a prostate cancer case treated with 70 Gy in 35 fractions, the imaging dose from CBCT is around 350 mGy and for the orthogonal kV imaging it is about 5 mGy^30^.

The second potential application could be combining the low-dose advantage with rapid reconstruction, to further enhance the precision of intra-fractional motion management and monitoring in modern treatment systems. The TrueBeam treatment system can acquire multiple kV projections from different angles around the patient^48–50^. This technique is commonly used for prostate cancer patients with gulden fiducials inserted into the prostate to monitor the target during treatment delivery. However, by utilizing our framework and these captured projections, clinicians might be able in the future to visualize volumetric anatomical changes in real-time during treatment delivery without additional imaging dose and need for fiducial markers. This capability would elevate intra-fractional monitoring from simple target monitoring to comprehensive 3D anatomical monitoring, potentially enabling more precise treatment adaptations that account for complex organ deformations and inter-relationships not visible in conventional 2D monitoring of target motion.

Notably, our anatomically informed loss function (ALF) significantly enhanced reconstruction quality in clinically relevant regions compared to conventional loss functions by allowing the model to focus on both general and clinically relevant information. While training with only the MAE and perceptual loss yielded acceptable global image metrics, they often failed to preserve image contrast, critical soft tissue interfaces, and anatomical boundaries essential for accurate radiotherapy treatments. The ALF’s framework prioritizes clinically important structures and represents a valuable methodological advancement for medical image synthesis applications using DL. Our framework could also be extended to other anatomical sites where organ motion and anatomical variations could impact treatment accuracy, such as lung, liver, and gynecological cancers. The image fusion-based approach and ALF loss function are generalizable concepts that could benefit various medical image synthesis tasks that deal with several and different imaging modalities.

Our findings extend previous work on DL-based image synthesis for radiotherapy in several important ways. While earlier studies have demonstrated the feasibility of generating synthetic CT from limited-angle reconstruction methods^26,32–44^, our fusion-based approach incorporates prior anatomical information from the planning CT, enabling more accurate reconstruction of anatomy while learning to extract viable anatomical information from 2D projections. To the best of our knowledge, there are no published similar frameworks utilizing this concept. Despite previous studies relied on developing patient-specific models (a unique model retrained for every patient case^33,34,43^, our framework allows the model to learn general anatomical representations and transform between imaging domains without the need for retraining for new patient cases.

Conversely, several limitations remain and warrant being addressed. First, our model was trained and evaluated using DRRs generated from CBCT images rather than actual kV projections acquired during treatment. While DRRs provide a reasonable approximation, real kV projections contain additional noise, scatter, and artifacts that might affect reconstruction quality. Nonetheless, for prostate cancer patients, our standard clinical protocols dictate differential imaging approaches based on patient characteristics. Patients with implanted fiducial markers typically undergo kV imaging only, while those without fiducials (our cohort, including post-prostatectomy and palliative cases) receive CBCT imaging exclusively. This protocol-driven limitation creates an inherent challenge in collecting matched pairs of CBCT and kV images from the same patients at the same timepoints. Hence, making it difficult to train and validate DL models using both modalities simultaneously. Future work should validate performance using clinical kV projections.

Further, our model accurately reconstructs most anatomical variations, but extreme changes in rectum gas or bladder filling (seen on the projections) occasionally resulted in reconstruction artifacts or amplification of bowel gas (Fig. 4a). This limitation is inherent to the sparse input data where certain complex 3D representations cannot be fully resolved from only two orthogonal projections. Incorporating additional oblique projections might improve reconstruction in these challenging cases. However, that will be at the cost of increased image dose and imaging time. Another challenge with DL models in general is regarding obtaining an accurate quantitative measure of uncertainty in the output prediction. That is to indicate poor performance and that there is a need for a real CBCT to be acquired due to high anatomical uncertainty.

Looking forward, our framework opens several promising avenues for clinical translation and further research. The most immediate application is integrating this approach into existing IGRT workflows, where a hybrid strategy might involve acquiring CBCT scans periodically (e.g., weekly) while using our framework for real-time 3D daily anatomy verification. Hence, reducing cumulative imaging dose and treatment time while maintaining treatment precision. Furthermore, future studies could assess whether treatment plans adapted using sCBCT provide comparable dosimetric outcomes to those using conventional CBCT.

The translational potential of our approach is particularly promising given the widespread of modern LINACs equipped with both 2D-kV and CBCT imaging capabilities. Thus, implementation of our sCBCT framework across radiotherapy clinics potentially requires no additional hardware modifications for wide adaptation. Furthermore, our framework is designed as an anatomy verification framework implemented after the couch shifts has been applied using the 2D-kV images to minimize introduction of alignment errors prior to treatment delivery.

In conclusion, our anatomically informed DL framework for generating real-time volumetric synthetic CBCT from orthogonal 2D projections and planning CT represents a promising step toward faster yet reliable anatomical verification in prostate cancer radiotherapy. It has the potential to provide faster support for clinical decision-making at a lower imaging dose to a large group of radiotherapy patients.

## Methods

### Patient data acquisition

This study was approved by the Regional Ethical Review Board in Lund (No. 2013/742) of Skåne University Hospital to retrospectively search our hospital database and collect patient data. All research conducted was performed in accordance with the relevant guidelines and regulations. As the study was a retrospective study without patient intrusion or intervention, all data were anonymized, so the authors did not directly obtain consent from each patient. The need for informed consent was waived by the Ethical Review Board at Skåne University Hospital. Retrospective image data from 419 prostate cancer patients treated at our clinic from 2019 to 2025 were collected and processed in this study. The patients received either 70 Gy in 35 fractions to the prostate bed (post-prostatectomy, salvage intention) or 36 Gy in 6 fractions to the prostate (palliative intention). For each patient, a pCT, a set of anatomical delineations (PTV, bladder, rectum, body) and multiple pre-treatment CBCTs acquired for patient alignment and anatomy verification were collected. All delineated structures were created in the treatment planning system Eclipse (Varian, Palo Alto, CA). The PTV structures were delineated by oncologists while the rectum and bladder were delineated by dosimetrists and approved by oncologists and medical physicists. The pCTs were acquired in supine position using a Siemens Somatom Definition AS plus (Siemens Medical Solutions, Erlangen, Germany) scanner with a 120-kVp spectrum, image resolution of 0.98 × 0.98 × 3.0 mm^3^, and sizes 512 × 512 × 150 up to 512 × 512 × 182, respectively. The pre-treatment CBCTs were acquired using the onboard kV system on our TrueBeam (Varian, Palo Alto, CA) LINACs with image resolution 1.98 × 1.98 × 3.0 mm^3^ and size of 512 × 512 × 88.

The orthogonal DRR projections were generated from each CBCT at projection angles of 0° and 270° using parallel projections, utilizing the open-source library CuPy (v.12.3.0). The DRRs were resized to 128 × 128 and had a resolution of 1.98 × 1.98 mm.

### Data preprocessing

All medical images were anonymized prior to data preprocessing. The preprocessing pipeline was implemented in two phases, phase 1 and 2 (Fig. 1b, Framework overview, Results). In phase 1, the preprocessing pipeline was implemented in Hero (v.2024.2.0) (Hero Imaging AB, Umeå, Sweden). The Preprocessing pipeline in Hero is provided below (see Code availability). To ensure consistent anatomical alignment, corresponding pCT and CBCT image pairs were rigidly registered and resampled using the CBCT parameters as reference. All images and radiotherapy structures were resized to 256 × 256 × 64 and multiplied by a binary body mask structure, derived from the external body contour delineated on pCTs. This step excluded irrelevant details located outside the patient’s body (e.g., CT and treatment couch), which might facilitate correct training of the models. Next, the processed images were saved in NIfTI format, in preparation for subsequent processing step.

The preprocessing in Phase 2 was implemented online during model training, where the data loading, preprocessing, augmentation, truncation of intensity values, and normalization were all performed on the fly using the open-source MONAI^45^ (v.1.3.2). framework. Data splitting was performed at the patient level during model training to prevent data leakage.

To optimize computational efficiency while preserving image resolution, all images were centrally cropped to 128 × 128 × 64. Subsequently, for the training dataset, random augmentation techniques including translations (± 5 pixels) and rotations (± 4°), were applied equally to the CBCTs and their corresponding radiotherapy structures. That is, to encourage robust anatomical feature mapping and mitigate overfitting. Image intensity values of the pCT and CBCTs were truncated between − 1000 and 2000 Hounsfield units (HU) and per case rescaled via min-max normalization to range [0, 1]. The delineated structures were converted into binary masks (0 or 1).

The DRRs were generated from the CBCT images after augmentation, to ensure that the anatomical information in the DRRs accurately reflected the augmented anatomy in corresponding CBCT. Next, all DRRs were normalized to a [0, 1] range using min-max normalization.

Finally, to further enhance computational efficiency and guide the model attention on clinically relevant regions, the sCT and CBCT images were multiplied by a volume binary mask of shape 128 × 128 × 64, retaining values of 1 within the center (128 × 64 × 64), and 0 elsewhere. This masking procedure permits elimination of irrelevant anatomical details outside the treatment region (e.g., femoral heads), while preserving the clinically relevant regions located at the image center (in axial view). Thus, we deal with cropped pCT, CBCT and sCBCT images in this framework instead of full field of view (Fig. 1b, Framework overview, Results).

### Model architecture

#### Fusion-Skip-Res model

Our model architecture, defined Fusion-Skip-Res, is a dual-branch encoder-decoder framework designed to synthesize volumetric sCBCT images by integrating information from dual orthogonal 2D-DRR projections and the patient’s 3D pCT (Fig. 2). The architecture comprises two encoding branches (2D encoder and 3D encoder), a feature fusion mechanism (transformation layer), and a decoding branch (3D decoder), that incorporates skip connections and residual blocks to enhance reconstruction quality.

The 2D encoder processes the input DRR projections to extract hierarchical semantic features representing the daily anatomical state captured by these views. This branch consists of a series of 2D convolutional blocks (Fig. 2b, Enconv-2D). Each Enconv-2D block contains a 2D convolutional layer (Conv-2D), followed by 2D instance normalization (InstaNorm-2D), a rectified linear unit (ReLU) activation, and another Conv-2D layer. In each block, the first Conv-2D layers utilize a kernel size of 3 × 3, stride of 2 × 2, and padding of 1 to progressively downsample the input while increasing feature channels. The last Conv-2D layers utilize kernel size of 3 × 3, stride 1 × 1, and padding of 1 to extract more image features post downsampling. This branch generates a latent vector encoding the daily anatomical state of the patient.

In parallel, the 3D encoder processes the volumetric pCT images to extract comprehensive 3D anatomical features that serve as a structural reference. This branch mirrors the 2D encoder, employing 3D convolutional blocks (Fig. 2b, Enconv-3D) containing initial Conv-3D layers (kernel size 3 × 3 × 3, stride of 2 × 2 × 2, and padding of 1). The last 3D instance normalization (InstaNorm-3D), ReLU activation, and final Conv-3D layers (kernel size of 3 × 3 × 3, stride 1 × 1 × 1, and padding of 1). This encoder branch generates a latent vector encoding the reference anatomical state of the patient captured for treatment planning.

Following the encoding stage, the latent representations from the 2D and 3D branches are combined through a feature fusion mechanism. The 2D latent vector is spatially reshaped to match the dimensionality of the 3D latent vector. These aligned features are then concatenated and processed by a 3D convolutional layer (Fig. 2b, Transform Layer) with a kernel size of 1 × 1 × 1, stride 1 × 1 × 1, and padding of 0. That is, to map the multi-modal (DRR and pCT) features into a shared latent space (the sCBCT space).

The fused features are then passed to the 3D decoder, which reconstructs the volumetric sCBCT through a series of upsampling steps. To facilitate efficient feature propagation and detail refinement, this branch incorporates two key enhancements: skip connections and residual blocks. Skip connections propagate feature maps from corresponding levels of the 3D encoder to the decoder, concatenating them before each decoding step (grey dashed lines, Fig. 2b). This helps preserve high-resolution anatomical details lost during downsampling. Furthermore, the decoder utilizes residual upsampling blocks (Fig. 2b, Res-Deconv-3D), where each block utilizes a trilinear upsampling layer followed by a residual connection around a set of Envond-3D layers. The final decoding block outputs the reconstructed sCBCT volume.

#### Model training

We implemented a standardized training protocol across all deployed models using Linux (v.20.04) operating system, Nvidia driver (v.525.85.05), CUDA (v.12.1), PyTorch^51^ (v2.3.0), and NVIDIA RTX 6000 Ada Generation GPUs. Prior to training, all model weights were initialized using the Kaiming He uniform method^52^.

For model parameter updates and optimization, the AdamW optimizer was utilized, configured with β₁ = 0.9, β₂ = 0.999, Ɛ = 10^− 8^, a weight decay of 10^− 2^, and an initial learning rate of 10^− 3^. All experiments were conducted with a batch size of one. Each model was trained for 40 epochs, with performance checking via masked metrics on the validation set at the end of each epoch. The learning rate decay schedule was deployed to reduce the learning rate in case of validation loss plateau by a factor of 10, if no improvement detected for 5 consecutive epochs and threshold 10^− 4^. The minimum allowed learning rate was set to 10^− 6^. Additionally, training checkpoints were utilized to save the best-performing model, defined as the one yielding the lowest validation loss on the validation set. The average model training times were approximately 11 h for the Fusion-Skip-Res model, and for the other models (available in Supplementary information) approximately 9 h for both the DRRs-only and Fusion models, and 10 h for the Fusion-Skip model.

#### Loss functions

As the primary loss function in this framework, we implemented a multi-component function combining both MAE (L_1_-norm) and PL applied on cropped sCBCT and CBCT images as follows:

1 $$\:{L}_{loss}=\alpha\:\:MAE+\beta\:\:P{L}_{(\text{3,8},\text{15,22}),}$$

Where MAE denotes the mean absolute error between the sCBCT and CBCT images:

2 $$\:MAE\:\left(sCBCT,CBCT\right)=\:\:\frac{1}{N}\:\sum\limits_{i}^{N}\:||sCBC{T}_{i}-CBC{T}_{i}||$$

α and $\:\beta\:$ are hyper-parameters representing weighting factors to balance the loss components (presented in Table 2, “Tables”), *i* is the pixel index, and PL_(3,8,15,22)_ is the perceptual loss defined as:

3 $$\:PL\:{\left(sCBCT,CBCT\right)}_{\text{3,8},\text{15,22}}=\:MSE(\:F{\left(sCBCT\right)}_{\text{3,8},\text{15,22}}\:,\:\:F{\left(CBCT\right)}_{\text{3,8},\text{15,22}}\:)$$,

Where 3, 8, 15, and 22 denotes the selected feature layers from a VGG19 network^53^, and MSE denotes the mean squared error loss between extracted image features:

4 $$\:MSE=\:\frac{1}{N}\:\sum\limits_{i}^{N}{\left(\:\:F{\left(sCBCT\right)}_{i}-\:F{\left(CBCT\right)}_{i}\:\right)}^{2},$$

and F represent the feature extraction function for chosen feature layers 3, 8, 15, and 22.

The PL entailed deploying a pretrained VGG19 network using the open-source Torchvision (v.0.19.1) library, initialized with IMAGENET1K_V1 weights, originally trained on image recognition tasks. The selection of feature layers was conducted based on recommendations by previous study^54^, to capture perceptually relevant features.

As the VGG19 model was initially developed for 2D RGB image recognition tasks, we implemented a 2.5D approach involving sequentially feeding in axial and sagittal slices of each sCBCT-CBCT pair to obtain a comprehensive PL representation. The images were converted to a pseudo-RGB input by duplicating them three times prior to calculation.

#### Anatomically informed loss function (ALF)

To explicitly guide the DL model to prioritize clinically relevant regions during training, we extended our primary loss function in Eq. (1) to incorporate anatomical information, forming ALF according to:

5 $$\:{L}_{ALF}=\alpha\:\:MA{E}_{1}+\beta\:\:P{L}_{1}+\gamma\:\:P{L}_{DRR}+\omega\:\:P{L}_{structures}\:$$

Where $\:MA{E}_{1}$ and $\:P{L}_{1}$ are defined in Eq. (1), $\:P{L}_{DRR}$ denotes PL calculated between the input DRRs and generated DRRs from the sCBCT images, and $\:P{L}_{structures}$ represents PL calculated solely at selected anatomical regions (PTV, bladder, and rectum).

**Fig. 6:**
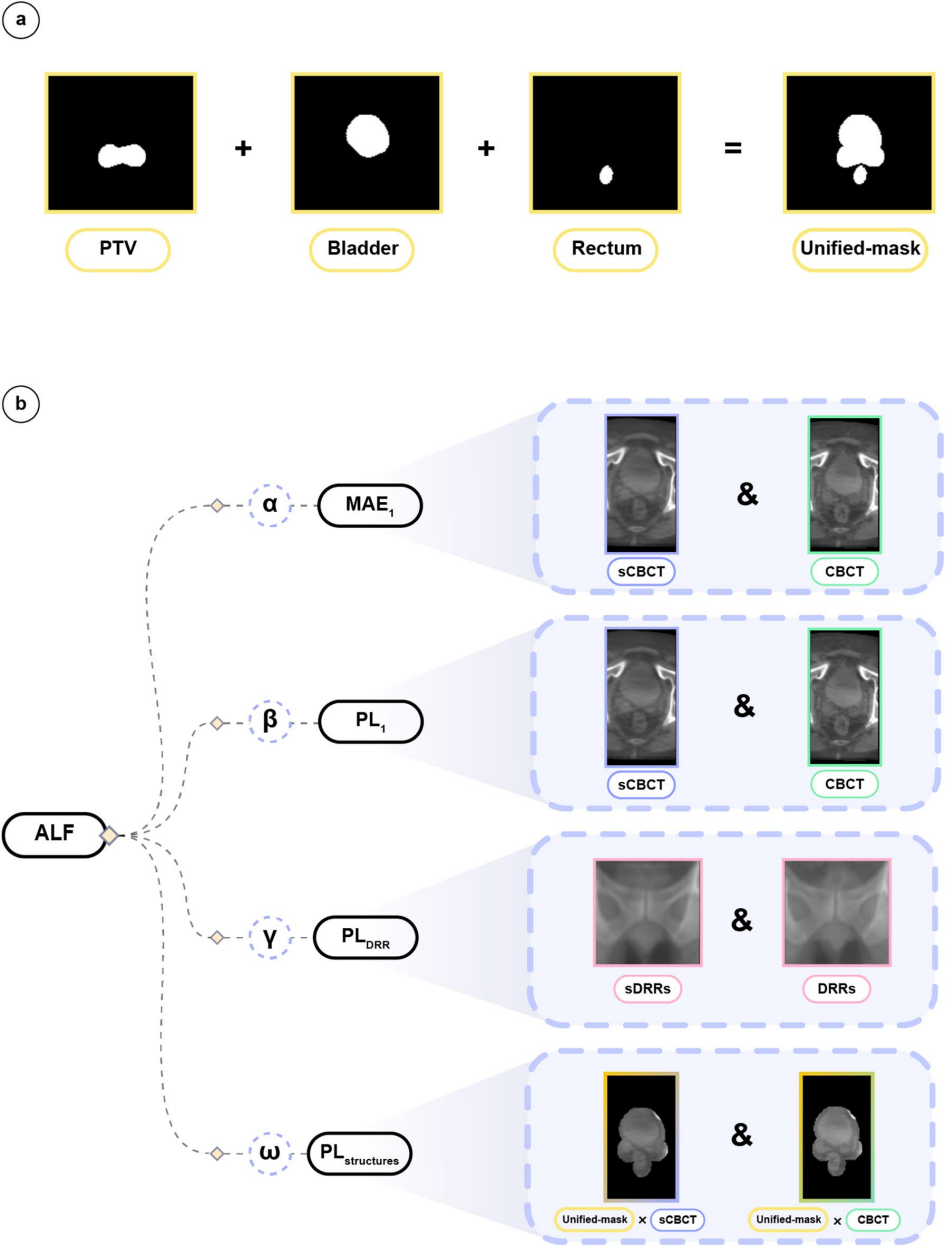
Overview of Unified-mask and ALF. (**a**) Representative samples of delineated structures (PTV, bladder, and rectum) as binary masks (yellow boxes) added together to form a binary unified mask, Unified-mask, used for ALF calculations in (**b**). (**b**) Visualization of the ALF multi-components along with their input data types and weighting factors described by Eq. (5). Here, sDRRs (pink, left) denote the DRRs (pink, right) generated from the sCBCT (purple) images to be compared with the DRRs generated from the ground truth CBCT (green).

Exclusively for the $\:P{L}_{structures}$, we construct a unified binary mask, Unified-mask (see Fig. 6a), by combining the binary masks of selected radiotherapy structures. Next the Unified-mask is expanded via dilation by three pixels to include more edge information. Finally, The Unified-mask is multiplied by the sCBCT/CBCT images to exclude all information outside the clinically relevant region (Fig. 6b). Moreover, $\:\alpha\:$, $\:\beta\:$, $\:\gamma\:$, and $\:\omega\:$ are weighting factors selected through grid search from 0 to 1 in steps of 0.01. The optimal weighting factors yielding best results by ALF (Figs. 3, 4 and 5, Results) are presented in Table 2.

#### Ablation experiments

To investigate the impact of loss function selection on sCBCT image quality (Fig. 5), we conducted a series of ablation experiments utilizing the best-performing model, Fusion-Skip-Res, as our baseline. Specifically, the model was retrained and evaluated on the test dataset using four distinct loss configurations: only MAE (onlyMAE), only PL (onlyPL), hybrid combination of MAE and PL (MAE&PL), and only ALF. The optimal weighting factors for each loss configuration are presented in Table 2 (Tables). Furthermore, for the MAE&PL loss, we conducted a grid search to identify the most effective values for the weighting factors $\:\alpha\:$ and $\:\beta\:$, ensuring that performance comparisons were not biased by suboptimal parameter choices. These experiments showcased the impact of each loss function on the overall reconstruction quality. Furthermore, we also implemented ablation experiments for different model architectures to find the most suitable architecture for our reconstruction task (Model ablation experiments, Supplementary information). The Fusion-Skip-Res was selected as it offered the best reconstruction image quality.

#### Evaluation metrics

Inspired by the SynthRAD2023 Grand Challenge framework^55^, we computed image similarity metrics within the body contour structure to fairly evaluate our DL models without including the background. Specifically, we adopted the following widely recognized metrics: mean absolute error (MAE), structural similarity index measure (SSIM), peak signal-to-noise ratio (PSNR), and learned perceptual image patch similarity (LPIPS)^31,56^.

The masked metrics, mMAE, mSSIM, and mPSNR were calculated using the open-source library, Scikit-image (v.0.21.0), as follows:

6 $$\:mMAE\:\left(x,y\right)=\frac{1}{||BODY||}\sum\limits_{i\in\:Body}||{x}_{i}-{y}_{i}||,\:$$

where $\:x$ and $\:y$ denotes the sCBCT and ground truth CBCT images, and BODY is the binary mask representing the patient body contour.

7 $$\:mSSIM\:\left(x,y\right)=\frac{1}{||BODY||}\:\sum\limits_{i\in\:Body}\frac{\left(2{\mu\:}_{x}^{i}{\mu\:}_{y}^{i}+{c}_{1}\right)\:\left(2{\sigma\:}_{xy}^{i}+{c}_{2}\right)}{\left({{\mu\:}^{i}}_{x}^{2}+{{\mu\:}^{i}}_{y}^{2}+{\left({k}_{1}L\right)}^{2}\right)\left({{\sigma\:}^{i}}_{x}^{2}+{{\sigma\:}^{i}}_{y}^{2}+{\left({k}_{2}L\right)}^{2}\right)\:\:}\:,$$

where *µ*^*i*^_*x*_ and *µ*^*i*^_*y*_ denote the local mean (luminance) of input images x and y, $\:{{\sigma\:}^{2}}^{i}$ and $\:{\sigma\:}^{i}$ are the local variance and covariance, respectively calculated within a $\:N\times\:N\times\:N$ window (with *N* = 7) centered on voxel *i*. *L* is the dynamic range of pixel values, *k*_*1*_ and *k*_*2*_ are constants set to 0.01 and 0.03, respectively.

The mSSIM metric calculates the structural similarity, contrast and luminance between images x, and y, yielding a score from 0 to 1, where high score indicates high image similarity. As for the mPSNR, it can be calculated according to:

8 $$\:mPSNR\:\left(x,y\right)=10\:{\text{log}}_{10}\left(\frac{{MAX}_{y}^{2}}{\frac{1}{||BODY||}{{\sum\:}_{i\in\:Body}({x}_{i}-{y}_{i})}^{2}\:}\right),\:$$

where $\:MA{X}_{y}$ is the maximum pixel value in ground truth CBCT image.

To further account for human-like perception of image similarity, we utilized LPIPS^57^ to quantify the perceptual similarity between sCBCT and CBCT images. LPIPS relies on computing the similarity between the activations of image patches, using a pretrained network. In this study, the predefined network was selected to be AlexNet^58^. Accordingly, instead of conducting a masked evaluation of LPIPS, we found it sufficient to directly utilize the cropped sCBCT/CBCT images as inputs. The cropped LPIPS (cLPIPS) evaluation was conducted using the open-source Torchmetrics (v.1.5.2) library. Lower cLPIPS scores (close to 0) indicate high perceptual image similarity.

The mSSIM metric quantifies the similarity in brightness, contrast, and structural features between the sCBCT and CBCT images within the body contour, providing a quantitative measure of perceptual image quality. The mSSIM metric returns a score ranging [0, 1], with values closer to 1 indicating a greater degree of similarity between the synthetic and ground truth image.

As for mPSNR, it is inversely proportional to MSE and can be used to evaluate image fidelity between corresponding pixel intensities in the sCBCT and CBCT images. It offers a quantitative measure of reconstruction accuracy given in decibels (dB). Higher mPSNR values indicate a lower error and greater fidelity of the reconstructed pixel intensity to the original image.

Together, these masked evaluation metrics offer a comprehensive evaluation of both the quantitative and perceptual quality of the reconstructed images with reduced influence of the background on their estimation.

## Supplementary Information

Below is the link to the electronic supplementary material.

Supplementary Material 1

## Data Availability

The collected, generated, or analyzed medical imaging data during this study are available from the corresponding author upon reasonable request.

## Code availability

All code for the Fusion-Skip-Res model, including training scripts and architectural definitions are available at https://github.com/MustafaKadhim/Anatomically-Infromed-Deep-Learning. The repository also contains (1) the full architecture of all comparison models used in our ablation studies (see Supplementary information), and (2) the Phase 1 data-preprocessing script implemented in Hero (v.2024.2.0.).

## Declarations

### Statistical information

To compare the impact of different loss functions on each image similarity metric in Fig. 5**c**, we first confirmed non-normality using the Shapiro–Wilk test (*p* < 0.05). We then applied the nonparametric Kruskal–Wallis test as an omnibus assessment of differences among groups, followed by Dunn’s post-hoc pairwise comparisons with Bonferroni correction to identify which pairs differed significantly. All analyses were performed in Python using SciPy (v.1.13.1) and Scikit-posthocs (v.0.11.4.).
