# Supplementary material for: Anatomically informed deep learning framework for generating fast, low-dose synthetic CBCT for prostate radiotherapy
Source: Sci Rep. 2025 Oct 15;15:36106. doi: 10.1038/s41598-025-23781-7 (PMC12528502; doi:10.1038/s41598-025-23781-7)
Supplement: Supplementary file 1 — Supplementary Material 1 [file 41598_2025_23781_MOESM1_ESM.docx]

**Supplementary information**

**Anatomically informed deep learning framework for generating fast, low-dose synthetic CBCT for prostate radiotherapy**

Mustafa Kadhim**^*^**^1,2^, Emilia Persson^2,3^, André Haraldsson^1,2^, Christian Jamtheim Gustafsson^2,3^, Mikael Nilsson^4^, Malin Kügele^1,5^, Sven Bäck^1,2^, Sofie Ceberg^1^

***** Corresponding author

^1^ Department of Medical Radiation Physics, Lund University, Lund, Sweden.

^2^ Radiation Physics, Department of Hematology, Oncology, and Radiation Physics, Skåne University Hospital, Lund, Sweden.

^3^ Medical Radiation Physics, Department of Translational Medicine, Lund University, Malmö, Sweden

^4^ Centre for Mathematical Sciences, Lund University, Lund, Sweden

^5^ Klinik und Poliklinik für Strahlentherapie, Universitätsmedizin Rostock, Rostock, Germany

Supplementary Notes 1-3

Supplementary Figures 1-3

Supplementary Notes

Supplementary Note 1: Model ablation experiments

We conducted ablation experiments for other model architectures, such as the DRRs-only, Fusion, and Fusion-Skip model, besides the Fusion-Skip-Res model to find the best model configuration for our task (See Investigated models, **Supplementary Note 2)**. To assess how model architecture impacts reconstruction quality of sCBCT images, we benchmarked performance across all different models on the test dataset using the ALF loss function. As observed (Fig. 3, **Supplementary Figures**), by altering the information flow in the encoders and decoder components of the model, different reconstruction quality and accuracy could be obtained in the sCBCT images. In all cases the Fusion-Skip and Fusion-Skip-Res models consistently outperformed the DRRs-only and Fusion models. As they yielded sCBCT images with fewer artefacts and higher visual fidelity to the ground truth CBCT images. Observed hallucination of anatomical structures can also be seen in the DRR-only model.

Supplementary Note 2: DRRs-only and Fusion models

The DRRs-only and Fusion models consist of a dual-branch encoder-decoder architecture, designed to integrate information from both 2D DRR projections and 3D pCTs to reconstruct 3D sCBCT images. The model comprises four key components: 2D encoder, 3D encoder, feature fusion, and a 3D decoder (see Fig.1, **Supplementary Figures**). The 2D encoder extracts semantic features from the DRRs to capture daily anatomical changes and combine this information with extracted anatomical features from the 3D encoder component of the pCT. The 2D encoder contains a series of 2D convolutional blocks (Enconv-2D, in Fig.1, **Supplementary Figures**), each including:

- A 2D convolutional layer (Conv-2D),
- 2D instance normalization (InstaNorm-2D) to stabilize training,
- And a rectified linear unit (ReLU) activation to introduce non-linearity.

Each Conv-2D layer in Enconv-2D implements:

- In features, out features, a kernel size of 3×3, stride 2×2, and a padding of 1.

The Enconv-2D blocks progressively downsampled the input while extracting hierarchical semantic features, ultimately compressing the DRR input into a latent representation we define as a latent vector, encoding daily anatomical changes in latent space.

In parallel, the 3D encoder processes volumetric pCT images to extract high-dimensional anatomical features, providing a comprehensive structural reference for the patient's anatomy. Its architecture mirrors the 2D encoder, replacing Enconv-2D, with its 3D counterparts Enconv-3D. The latent vector generated by the 3D encoder contains essential 3D anatomical representations required for reconstructing the patient's anatomical state as observed during the treatment planning phase (captured by pCT).

Following the generation of latent vectors from the 2D and 3D encoders, the latent vector from the 2D encoder is reshaped to match the dimensionality of the 3D encoder’s output. These vectors are then concatenated and passed through a transformation layer (Fig.1**, Supplementary Figures**) implementing a single 3D convolutional (Conv-3D) layer with kernel size of 1×1×1, stride 1×1×1, and a padding of 0. This layer maps the combined features into a shared latent space analogous to the CBCT latent space.

The output of the transformation layer is subsequently passed to the 3D decoder, which decompresses the transformed latent vector through a series of upsampling blocks (Deconv-3D). These blocks employ:

- A trilinear upsampling layer (upscale factor 2),
- Conv-3D,
- 3D instance normalization (InstaNorm-3D),
- And ReLU activation.

The last Deconv-3D block in the decoder implements an upscale factor of 1. Each Conv-3D layer in the Deconv-3D block implements:

- In features, out features, a kernel size of 3×3×3, stride 1×1×1, and a padding of 1.

For the DRRs-only model, the latent vector of the 3D encoder is multiplied by zero to eliminate any propagation of anatomical features from the pCTs to the decoder. Therefore, no modifications of architecture were needed between the DRRs-only and Fusion models.

Supplementary Note 3: Fusion-Skip model

To enhance anatomical detail retention from the pCT during hierarchical feature downsampling, we extended the Fusion model with skip-connections implemented between the 3D encoder and decoder branches (Fig 2, grey dashed arrows, **Supplementary Figures**). The skip-connections propagate features from the 3D encoder’s Enconv-3D blocks to selected Deconv-3D blocks in the decoder. The features are then merged (concatenated) with features processed by the previous decoder layer and forwarded to subsequent decoder blocks.

Supplementary Figures


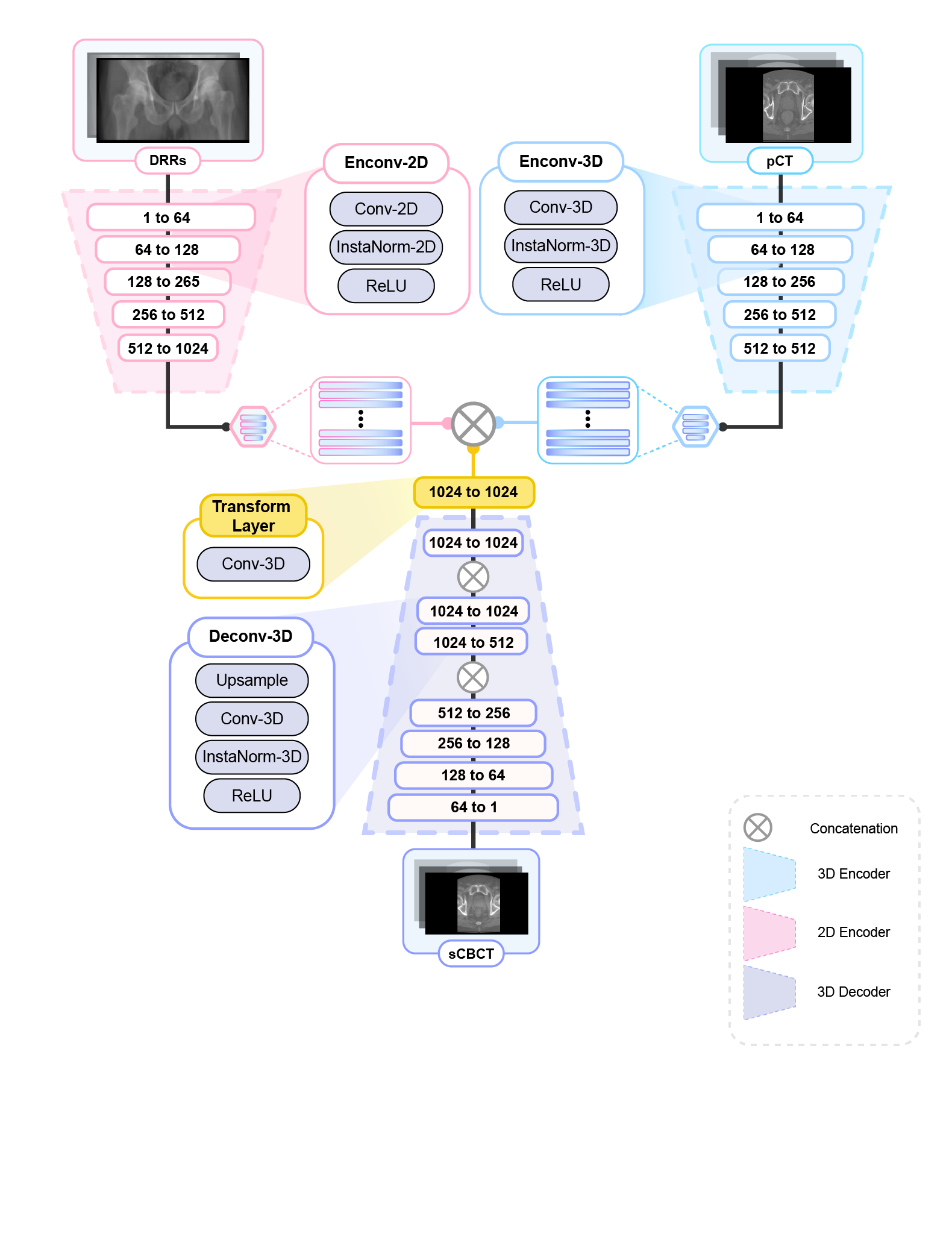


**Fig. 1: DRRs-only and Fusion models architectures**: In the DRRs-only model, only the features extracted by the 2D encoder (pink) branch are forwarded to the 3D decoder (purple), enabling sCBCT reconstruction exclusively from DRRs. As for the Fusion model, both the 2D encoder and 3D encoder (blue) branches contribute to the reconstruction of the sCBCT image.


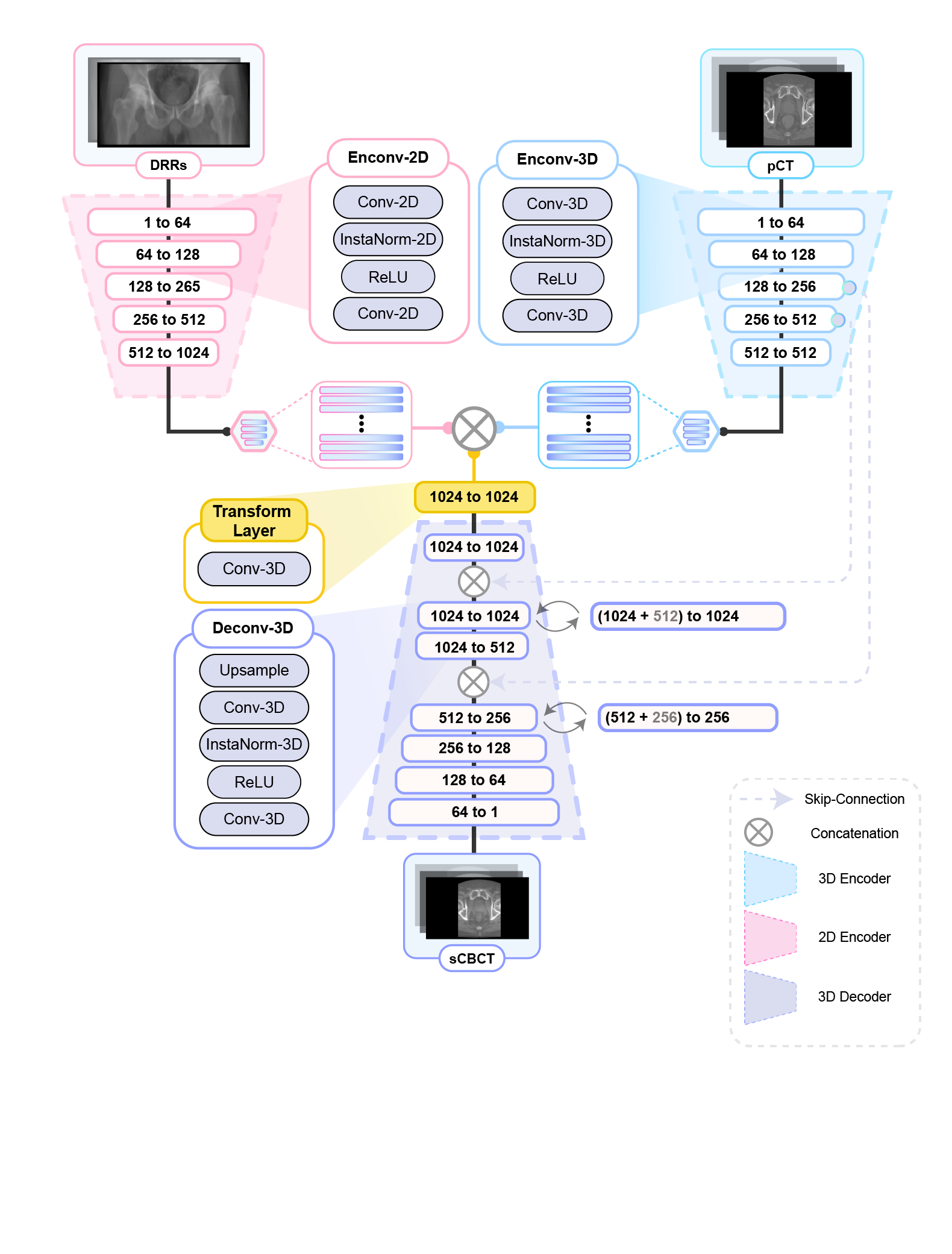


**Fig. 2: Overview of Fusion-Skip model architecture:** *modifications of the Fusion model by incorporation of skip connections (grey arrows) and additional convolutional blocks. The decoder architecture was adjusted (blocks with gray circular arrows) to manage the increased number of input features following concatenation.* *For the Fusion-Skip model, an additional convolutional layer (Conv-2D/Conv-3D) was integrated into both the encoders and the decoder blocks to enhance feature extraction and representation.*


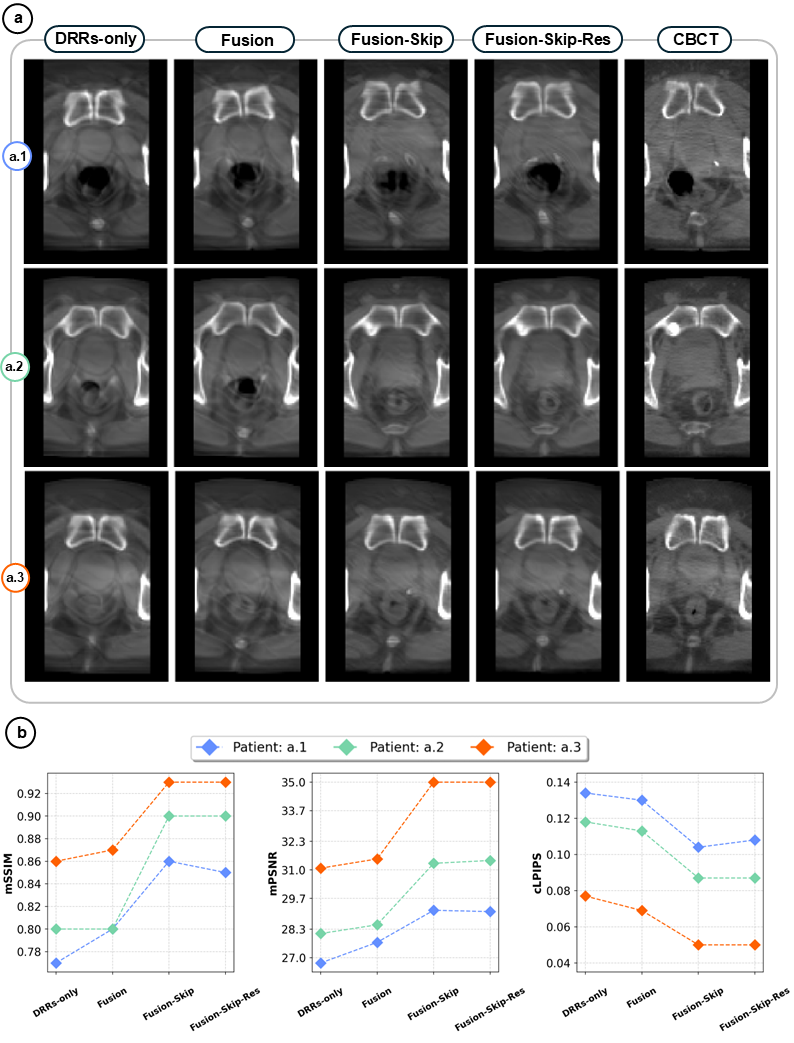
**Fig. 3: sCBCT Image quality depends on selected model architecture. a)** sCBCT reconstructions of representative test samples (rows, a.1, a.2, a.3) shown for each explored model architecture (columns). **b**) Quantitative evaluation of the mSSIM, mPSNR, and cLPIPS metrics calculated for each patient in (**a**) to demonstrate the patient-specific influence of model architecture on image quality.
